# Certainty of evidence assessment in high‐impact medical journals: A meta‐epidemiological survey

**DOI:** 10.1002/cesm.70014

**Published:** 2025-03-19

**Authors:** Madelin R. Siedler, Neha Tangri, Leena AlShenaiber, Tejanth Pasumarthi, Faisal Shaukat Ali, Volf Gaby, Katie N. Harris, Yngve Falck‐Ytter, Reem A. Mustafa, Shahnaz Sultan, Philipp Dahm, M. Hassan Murad, Rebecca L. Morgan

**Affiliations:** ^1^ Evidence Foundation Cleveland Heights Ohio USA; ^2^ Department of Kinesiology and Sport Management Texas Tech University Lubbock Texas USA; ^3^ College of Medicine and Health Sciences Khalifa University Abu Dhabi UAE; ^4^ School of Interdisciplinary Science, McMaster University McMaster University Hamilton Ontario Canada; ^5^ Department of Gastroenterology, Hepatology and Nutrition The University of Texas Health Science Center Houston Texas USA; ^6^ Department of Health Research Methods, Evidence, and Impact (HEI) McMaster University Hamilton Ontario Canada; ^7^ School of Medicine Case Western Reserve University Cleveland Ohio USA; ^8^ Division of Gastroenterology University of Minnesota Minneapolis Minnesota USA; ^9^ Division of Urology University of Minnesota Minneapolis Minnesota USA; ^10^ Mayo Clinic Evidence‐Based Practice Center, Mayo Clinic Rochester Minnesota USA

**Keywords:** certainty of evidence, evidence‐based medicine, GRADE, systematic reviews

## Abstract

**Introduction:**

While certainty of evidence assessment is key to a rigorous and transparent systematic review, it is unknown how – and how frequently – it is assessed in systematic reviews. The objective of this study was to examine the prevalence and approaches used for certainty of evidence assessment in systematic reviews published in high‐impact medicine journals over the past 11 years.

**Methods:**

A PubMed search and hand‐searching of relevant journal websites identified systematic reviews published between 24 January 2013 and 23 January 2024 in any of the ten highest‐impact journals in the General and Internal Medicine category of the Journal Citation Report. Two reviewers independently selected any systematic review related to health outcomes assessing certainty of evidence using any method. We extracted data related to review characteristics, certainty of evidence and risk of bias/methodological quality assessment frameworks, and reported consideration of certainty of evidence domains. Logistic regression examined year of publication to determine whether the prevalence of certainty of evidence assessment changed over time.

**Results:**

Of 1,023 included reviews, 346 (33.8%) assessed certainty of evidence. Prevalence of certainty of evidence assessment increased over time (0.16 ± 0.2; *p* < .001). Most (89.3%) of reviews used the Grading of Recommendations Assessment, Development and Evaluation (GRADE) framework to assess certainty of evidence.

**Conclusion:**

Only one in three systematic reviews published in the highest‐impact medical journals over the past 11 years assessed certainty of evidence, though prevalence increased over time. The use of specific domains within each certainty of evidence framework was not clearly described in all reviews.

## 1 INTRODUCTION

The assessment of the certainty of evidence has been considered a key function of a systematic review [1] and is also a distinct reporting standard for systematic reviews [2], though systems of grading the evidence long predate this guidance [3]. Certainty of evidence assessment indicates to evidence users a level of trustworthiness that is warranted for each estimate provided in the systematic review and also provides an indication of where the true effect lies in relation to a threshold or range [4]. As described in greater detail elsewhere [5], certainty of evidence assessment extends beyond the examination of each study's methodological quality or risk of bias by considering additional methodological domains such as imprecision and inconsistency [4] to arrive at an overall judgment of the trustworthiness of presented evidence across studies. A number of different approaches for assessing certainty of evidence have been proposed and used in the literature. Common examples include the Grading of Recommendations Assessment, Development, and Evaluation (GRADE) framework [3], the United States Preventive Services Task Force (USPSTF) system [6], and the Oxford Center for Evidence‐Based Medicine (OCEBM) Grades of Recommendation [7].

Although the 2020 update of the Preferred Reporting Items for Systematic reviews and Meta‐Analyses (PRISMA) statement includes a standalone item for reporting the use of a framework to assess certainty of evidence [8], it is unclear how frequently certainty of evidence is assessed in systematic reviews in the field of general and internal medicine and what approaches are most frequently taken for this process. A 2016 convenience sample of systematic reviews published by four major medical journals, the Cochrane Collaboration, and the Agency for Healthcare Research and Quality (AHRQ) Evidence‐based Practice Center reported that approximately 55% of the included reviews assessed certainty of evidence. However, this assessment did not consider all systematic reviews published in the top‐ranked medical journals and was limited to only the most recent ten reviews from each source. Therefore, the objective of this study was to systematically examine and describe the frequency and methods with which certainty of evidence is assessed in systematic reviews published in high‐impact journals, which usually have large readership and reach and which provide evidence that directly affects patient care.

## 2 MATERIALS AND METHODS

This report adheres to the published reporting guidelines for meta‐epidemiological research [9] and the PRISMA 2020 guidance [2].

### 2.1 Data sources and searches

Methods were similar to those of a previously published study examining the frequency and nature of certainty of evidence assessment in the sports science literature [5]. In brief, we searched PubMed for any systematic review or meta‐analysis published between 24 January 2013 and 23 January 2024 in any of the ten highest‐impact journals in the General and Internal Medicine category as per the 2021 data from the Journal Citation Report. The time period for this search was originally developed to allow for a full ten‐year span of recent systematic reviews to be represented. This search was updated 1 year later, resulting in an eleven‐year window of articles assessed. The search strategy is presented in Supplemental Material 1. All but one of the ten journals of interest were indexed in PubMed for the entire specified time period. The one remaining journal (*Nature Reviews Disease Primers)* was not indexed in PubMed before 2016. In this case, two authors manually screened each abstract within the relevant timeframe via the journal website.

### 2.2 Study selection

Using Covidence systematic review software (Veritas Health Innovation, Melbourne, Australia), pairs of authors independently screened titles and abstracts of records for any systematic review, and then screened full texts for any systematic review that assessed the certainty of evidence in any way. All articles that self‐identified in the title or abstract as a systematic review were included. In addition, articles that reported applying key components of systematic review methodology (e.g., adherence to the PRISMA guidelines, the use of multiple databases to compile records, and the independent screening of titles/abstracts and full reports) were included for further consideration, with consensus between authors achieved as needed. Meta‐analyses that were not based on a systematic review of evidence were not included.

Certainty of evidence assessment was defined as the evaluation and consideration of two or more conceptual domains that can affect the confidence in a reported effect estimate (e.g., risk of bias/study limitations, inconsistency/heterogeneity, imprecision/sample size, etc.). Reviews that did not evaluate a question related to health outcomes (e.g., reliability/validity studies, non‐comparative prevalence reviews, cost reviews, etc.) were excluded, as were reviews that included other systematic reviews (e.g., overviews, umbrella reviews, or systematic reviews not limited to primary research in their search and/or analyses). Screening conflicts were managed via third‐person tie break at the title/abstract stage and via consensus at the full‐text stage.

### 2.3 Data extraction

Data for all 346 reviews that assessed certainty of evidence were extracted in Google Sheets (Alphabet Inc., Mountain View, CA, USA) by a primary reviewer and reviewed by a secondary reviewer. Conflicts at the data extraction stage were resolved via consensus or with consultation of the first author (MRS) when necessary. Data extracted included review characteristics (year published, journal of publication, global region of corresponding author, content area of review, number and types of studies included [randomized vs. non‐randomized studies], and question type [intervention/exposure, prognosis, or diagnosis]); whether quantitative meta‐analysis was undertaken for at least one outcome; whether a funding statement was included; tools used to assess risk of bias or methodological quality; systems used to assess certainty of evidence, if reported; whether certainty of evidence was assessed at the outcome level (i.e., not only at the individual study level); and whether practice recommendations were included. Additionally, the conceptual domains used to assess certainty of evidence were noted. The specific domains used to assess certainty of evidence may vary across reviews that purportedly use the same framework [5]. Therefore, conceptual certainty of evidence domains were only marked as reported if explicitly described in the methods or evaluated in the results of the review (e.g., within a Summary of Findings or Strength of Evidence table).

### 2.4 Data synthesis and analysis

Descriptive data were presented as median and inter‐quartile range (IQR) unless otherwise reported. Year of publication was used as a predictor variable within a logistic regression to determine the effect of publication time on the prevalence of certainty of evidence assessment. For this analysis, the entire population included all analyzed articles as well as any systematic review that was otherwise eligible (e.g., examined a health‐related research question) but did not assess certainty of evidence. Analyses were conducted in RStudio (version 2023.12.1 + 402) using the R software (version 4.3.1, R Foundation for Statistical Computing, Vienna, Austria).

## 3 RESULTS

**Figure 1:**
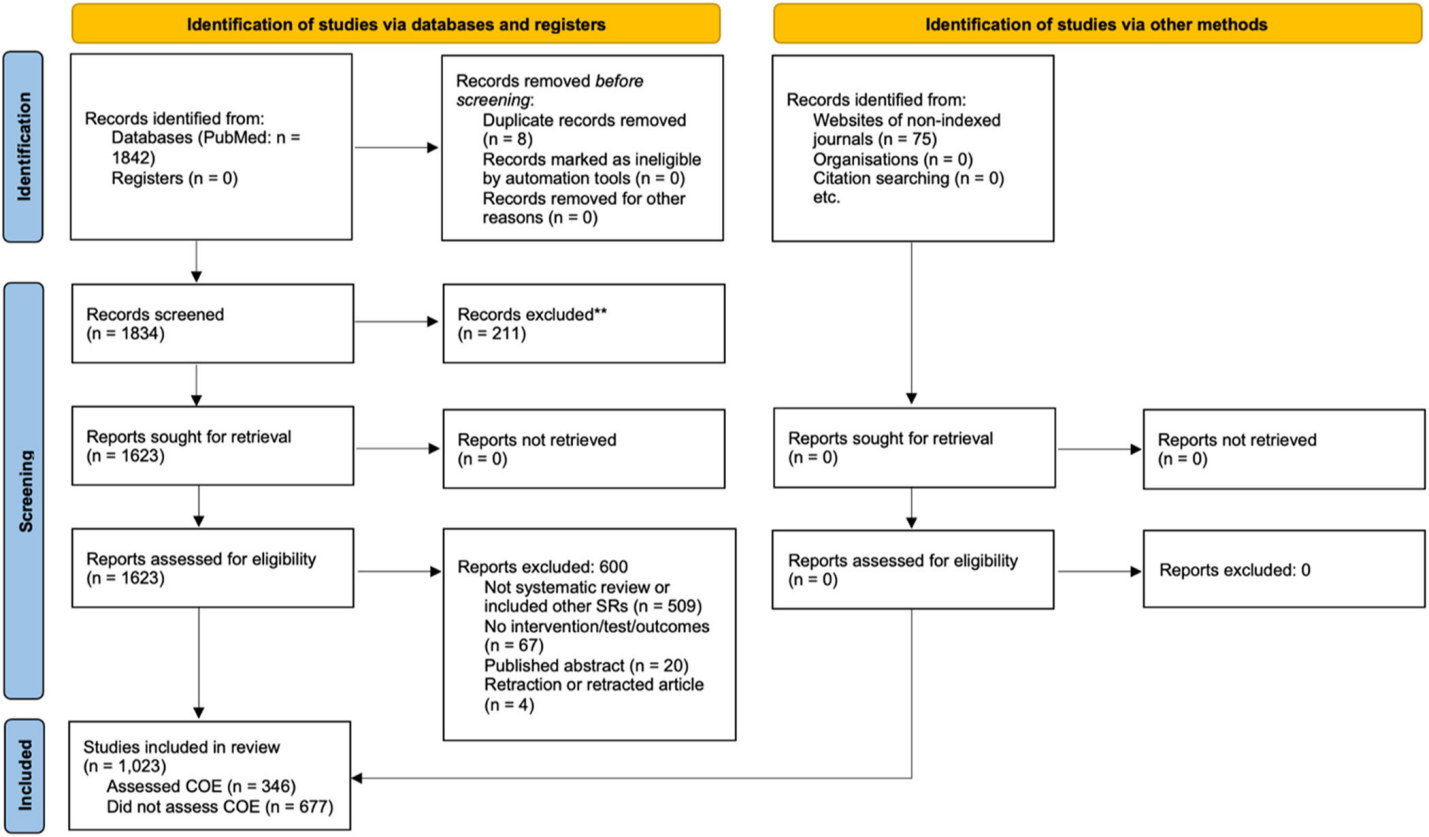
PRISMA 2020 flow chart.

A total of 1,917 records (1,842 articles indexed in PubMed and an additional 75 located via manual search) were screened. Eight duplicates were removed with an additional 286 records (211 from PubMed and 75 identified via manual search) excluded at the title and abstract screening stage. At the full‐text screening stage, 1,023 articles were ultimately included out of 1,623 screened. Of these, 346 of these were deemed to have assessed certainty of evidence and were further analyzed. A detailed PRISMA flowchart with reasons for exclusion at the full‐text stage is presented in Figure 1.

### 3.1 Characteristics of certainty of evidence assessment

Of the 1,023 eligible systematic reviews included in this analysis, 346 (33.8%) reported assessing certainty of evidence and were examined for analysis. Characteristics of the 346 reviews that assessed certainty of evidence are presented in Table 1. These reviews included a median of 35.5 studies (IQR: 19–67.75). In 11 reviews, the number of randomized controlled trials (RCTs) and/or non‐randomized studies (NRS) was unreported or unclear. Of these, the type of study included was not reported in four reviews and was reported as a mix of RCTs and NRS in seven. Of the remaining 119 reviews including a mix of RCT and non‐randomized study designs, a median of 16 (5–30.5) RCTs and 16 (6–37.5) NRS were included. As shown in Table 1, 13% of reviews that assessed certainty of evidence included a practice recommendation statement aimed at clinicians.

**Table 1:** Characteristics of included systematic reviews.

Characteristics of included systematic reviews (*n* = 346)	Total (%)
***Journal***	
*Annals of Internal Medicine*	115 (33.2)
*Journal of the American Medicine Association (JAMA)*	94 (27.2)
*British Medical Journal (BMJ)*	93 (26.9)
*Lancet*	20 (5.8)
*JAMA Internal Medicine*	19 (5.5)
*Lancet Digital Health*	3 (0.9)
*Journal of Travel Medicine*	2 (0.6)
*Military Medical Research*	0 (0)
*Nature Reviews Disease Primers*	0 (0)
*New England Journal of Medicine*	0 (0)
***Year Published***	
2013	25 (7.2)
2014	14 (4.0)
2015	21 (6.1)
2016	34 (9.8)
2017	26 (7.5)
2018	37 (10.7)
2019	39 (11.3)
2020	37 (10.7)
2021	30 (8.7)
2022	51 (14.7)
2023‐24	32 (9.2)
***Region*** **(Note: Some articles indicated multiple regions)**	
North America	236 (68.2)
Europe	62 (17.9)
Asia	25 (7.2)
Australia & New Zealand	23 (6.6)
South America	2 (0.6)
Africa	0 (0.0)
***Content Area***	
Cardiology	48 (13.9)
Pain medicine	25 (7.2)
COVID‐19	22 (6.4)
Infectious disease	21 (6.1)
Nutrition	21 (6.1)
Reproductive health/Obstetrics/Gynecology	19 (5.5)
Endocrinology	17 (4.9)
Neurology	17 (4.9)
Pulmonology	13 (3.8)
Metabolic disease	12 (3.5)
Public health	12 (3.5)
Mental health	11 (3.2)
Other (all other areas with *n* < 10)	108 (31.2)
***Question Type*** **(Note: Some articles included multiple question types)**	
Intervention/exposure	294 (85.0)
Diagnosis	54 (15.6)
Prognosis	19 (5.5)
***Types of Studies Included***	
Randomized controlled trials only	171 (49.4)
Non‐randomized studies only	41 (11.8)
Mix of randomized and non‐randomized studies	130 (37.6)
Not reported/unclear	4 (1.2)
***Conducted meta‐analysis for at least one outcome***	
Yes	277 (80.1)
No	69 (19.9)
***Assessed certainty of evidence on an outcome/predictor level***	
Yes	340 (98.3)
No/Unclear	6 (1.7)
***Included recommendation(s)***	
Yes	45 (13.0)
No	301 (87.0)
***Included funding statement***	
Yes	331 (95.7)
No	15 (4.3)

**Figure 2:**
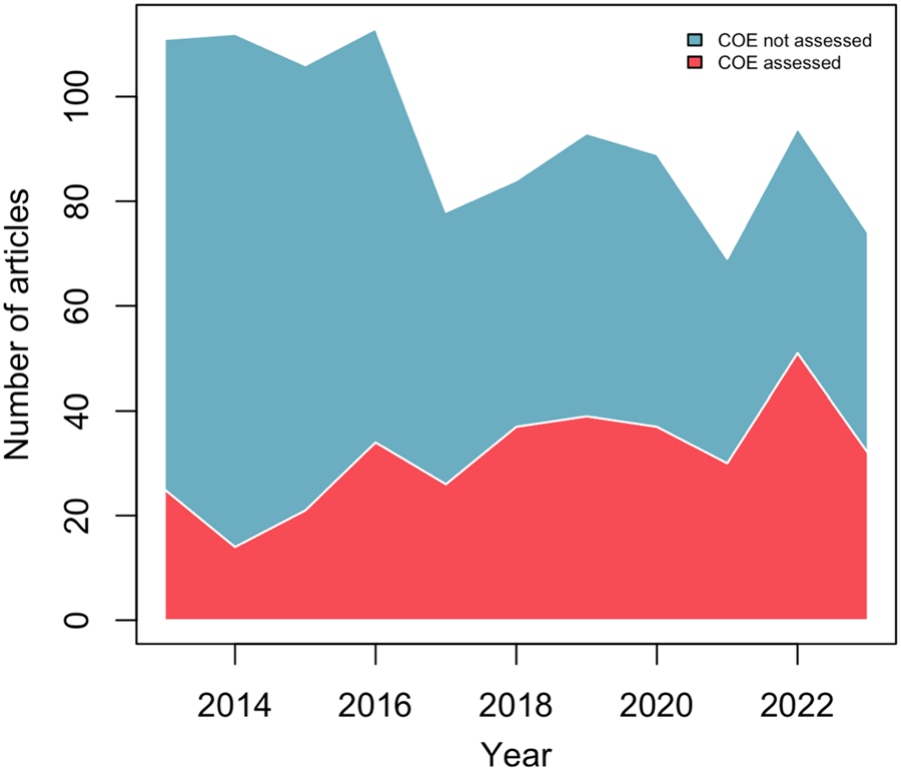
Area plot of certainty of evidence assessment in systematic reviews over time. COE: certainty of evidence.

None of the eligible reviews published in *Military Medical Research* assessed certainty of evidence. The two remaining journals (the *New England Journal of Medicine* and *Nature Reviews Disease Primers*) included no eligible systematic reviews for analysis. The prevalence of certainty of evidence assessment (i.e., percent assessing certainty of evidence out of all eligible reviews) ranged from 12.5% of all eligible reviews published in 2014 to 54.3% of those published in 2022. Furthermore, year of publication was a statistically significant predictor of whether an otherwise eligible systematic review assessed certainty of evidence (coefficient: 0.16 ± 0.02; *p* < .001), indicating that the prevalence of certainty of evidence assessment increased over the time period captured (Figure 2).

### 3.2 Risk of bias/methodological quality assessment

Systematic review authors reported assessing risk of bias and/or methodological quality of included studies in all but one (99.7%) of the 346 examined systematic reviews. The most commonly used tools to assess risk of bias included those by the Cochrane Collaboration (e.g., Risk of Bias 2 [10] for RCTs and Risk of Bias In Non‐randomized Studies of Interventions [ROBINS‐I] [11] for NRS), with 206 (59.5%) reviews indicating use of one or more of these tools. The USPSTF Criteria for Assessing Internal Validity of Individual Studies [12] was cited in 67 (19.4%) reviews. While the AHRQ Methods Guide for Effectiveness and Comparative Effectiveness Reviews [13] was cited in 32 (9.2%) reviews, it should be noted that this guide does not provide specific criteria for methodological quality assessment but provides features of acceptable tools that are already available. However, some reviews citing the use of the AHRQ guide described detailed methods in which a “study limitations” assessment was based solely on study design (e.g., population‐based observational cohort studies as “low,” single‐institution, small cohort studies, or case‐control studies as “medium,” and all other study designs as having “high” study limitations). Other frequently cited tools included the Newcastle‐Ottawa Scale [14] for NRS designs (6.6%) and the QUADAS tool [15] used for quality assessment of diagnostic accuracy studies (5.2%). The use of various customized tools was reported in 12 (3.5%) reviews [16, 17, 18, 19, 20, 21, 22, 23, 24, 25, 26, 27].

### 3.3 Certainty of evidence assessment frameworks

The most commonly used framework for assessing certainty of evidence was GRADE (89.3%). This includes GRADE adaptations to network meta‐analyses—that is, Confidence in Network Meta‐Analyses (CINeMA)—as well as adaptation by AHRQ Evidence‐based Practice Program [28]. The USPSTF approach, which uses various components of GRADE, was cited in 33 reviews (9.5%). Less commonly cited systems included the American Heart Association Levels of Evidence [29], the OCEBM Grades of Recommendation criteria [7], and a custom framework partially based on GRADE [30] being used by only one to two reviews each. Table 2 presents each framework with its prevalence in our population and specific approach used for assessing certainty of evidence.

**Table 2:** Approaches cited for assessing certainty of evidence and their conceptual domains.

Framework	Certainty of evidence levels	Number (%) of all reviews (*n* = 346)	Conceptual domains Included in framework (% of reviews explicitly reporting consideration of each domain)
GRADE [3]	High Moderate Low Very Low	209 (60.4)	Risk of bias/Methodological quality (91.4) Indirectness (77.0) Inconsistency (84.7) Imprecision (86.6) Publication bias (61.7) Magnitude of effect (9.6) Dose‐response gradient (7.2) Plausible residual confounding (5.7) *Coherence – when applied to NMAs* (11.5)
GRADE adaptation in AHRQ Methods Guide [28]	High Moderate Low Insufficient	100 (28.9)	Risk of bias/Methodological quality (91.0) Consistency (93.0) Precision (90.0) Directness (76.0) Reporting – includes publication bias, selective outcome reporting, and selective analysis reporting (42.0) Magnitude of effect (6.0) Dose‐response gradient (2.0) Plausible residual confounding (3.0)
USPSTF [6]	Key Question Level Convincing Adequate Inadequate Certainty of Net Benefit High Moderate Low	33 (9.5)	Internal validity – Study design/Risk of bias/Methodological quality (90.9) External validity – Directness/applicability (84.8) Consistency (100) Precision (84.8) Coherence – when considering linkages across evidence to assess net benefit (0)
AHA [29] Levels of Evidence	A B C	2 (0.6)	Study design (100.0) Precision (100.0)
OCEBM Grades of Recomm‐endation [7]	A B C D	1 (0.3)	Study design (100.0) Consistency (100.0) Directness (0)

Abbreviations: AHA, American Heart Association; AHRQ, Agency for Healthcare Research and Quality; GRADE, Grading of Recommendations Assessment, Development and Evaluation; NMA, Network Meta‐Analysis; OCEBM, Oxford Centre for Evidence‐Based Medicine; USPSTF, United States Preventive Services Task Force.

Of the 324 articles explicitly describing the consideration of at least one conceptual domain within the related certainty of evidence framework, these mainly comprised study design/risk of bias/methodological quality (97.2%), consistency (94.1%), and precision (93.5%) of the evidence. Consideration of directness/applicability (82.1%) and publication bias (58.6%) was also frequently reported. Less often, reviewers explicitly reported using magnitude of effect (8.3%), incoherence/intransitivity (7.7%), presence of a dose–response gradient (5.9%) and plausible residual confounding (4.6%). Twenty‐two (6.4%) of the reviews did not explicitly mention the use of any specific domains to assess certainty of evidence in their methods or results.

## 4 DISCUSSION

### 4.1 Key findings

This meta‐epidemiological survey reports on the state of certainty of evidence assessment in systematic reviews published in high‐impact journals in the field of general and internal medicine. Notably, the present review demonstrates that over the past 11 years, only about one‐third (33.8%) of eligible systematic reviews assessed certainty of evidence. However, the prevalence of certainty of evidence assessment appears to be increasing over time, with over half (54.3%) of eligible reviews published in 2022 assessing certainty of evidence. Year of publication was a statistically significant predictor of certainty of evidence assessment. Furthermore, the majority of systematic reviews assessing certainty of evidence adhered to an important PRISMA standard for reporting “sources of financial or nonfinancial support for the review, and the role of the funders or sponsors in the review” [2], with 95.7% of reviews including a funding statement. A similarly large majority (98.3%) of systematic reviews assessed certainty of evidence on the level of outcomes or predictors (Table 1).

Considerations of the quality, consistency, and quantity (i.e., precision) of evidence have been proposed as the minimum criteria for any approach for assessing certainty of evidence [31]. Nearly all of the systematic reviews examined in our population applied frameworks meeting this standard (Table 2). The most frequently cited framework for certainty of evidence assessment was GRADE (89.3%). The USPSTF system uses many of the same domains as GRADE but excludes a consideration of publication bias. The American Heart Association and OCEBM systems, which were used much less frequently in our population, rely mainly on study design (with less emphasis on risk of bias or methodological quality) and precision (typically operationalized as number of studies without evaluation of the actual uncertainty in the pooled estimate). The OCEBM Grades of Recommendation system further takes directness into account by penalizing bodies of evidence informed by extrapolations from higher‐level studies [7].

The range of conceptual domains applied within a framework in theory can differ largely from their application in practice. In the current population, systematic review authors were more likely to explicitly report considering study quality, consistency, and precision than other domains included in commonly used systems such as directness/applicability and publication/reporting bias. Methodological domains introduced by GRADE to rate certainty up were much less commonly cited (large magnitude of effect, presence of a dose–response gradient, and plausible residual confounding that would be expected to increase confidence in the observed effect). This may be due to the fact that these domains are considered in non‐randomized studies, which were less common in these reviews published by high‐impact journals. An alternative explanation may be that these domains are less well‐known or understood by systematic reviewers.

Our results demonstrate that the reported use of a given certainty of evidence assessment framework does not guarantee the application of each domain within that framework; at the very least, it does not guarantee that these domains will be thoroughly described in the methods or results of each review. The exclusion of certain domains may be the result of a deliberate decision by authors to forego consideration of a certain domain. For instance, authors may choose not to assess publication bias if a small number of studies precludes the generation of a funnel plot and related statistical tests; however, these authors may be unaware that there are alternative ways to assess publication bias in such cases. Improved thoroughness of reporting by systematic review authors about how certainty of evidence was specifically assessed – including all domains within the framework considered and which, if any, were deliberately disregarded – will help clarify the nature of this underlying issue. In other cases, it is possible that authors implicitly considered certain domains but did not transparently describe their process in the methods or results.

A minority (13.0%) of systematic reviews within our population included language that could be interpreted as a clinical practice recommendation (e.g., statements using wording such as “clinicians should…” or “it is recommended…” when describing the key conclusions of a review). The formulation of clinical practice recommendations entails additional considerations such as variability in values and preferences, resource use, and the balance between benefits and harms [32] as well as input from clinical experts, patients, and other key stakeholders. It should thus be noted that systematic reviews should avoid using language that may be interpreted as a recommendation outside of the context of a clinical guideline document.

### 4.2 Relation to previous research

A similar survey described certainty of evidence assessment in systematic reviews published in high‐impact sports science journals [5]. That study similarly demonstrated that a minority (20.6%) of eligible systematic reviews published over a 6‐year timeframe assessed certainty of evidence. Our findings are also in line with a contemporary examination of the physical rehabilitation literature by Gianola and colleagues, which found that 29% of all reviews published in 2020 assessed certainty of evidence [33]. A 2016 investigation by Kane et al. [34] found that 55.3% of systematic reviews published in the *Annals of Internal Medicine*, *BMJ, JAMA*, and *Pediatrics* or as reports from the Cochrane Collaboration or AHRQ used a systematic rating system. This figure is generally in line with our findings that together, the former three journals demonstrated a mean certainty of evidence assessment rate of 40.5% over our eleven‐year timeframe.

The prevalence of certainty of evidence assessment in the sports science literature appeared to be increasing over time [5], although the relationship was weaker than in the current investigation (0.07 ± 0.04; *p* = .05). Additionally, the plurality of GRADE among all reviews assessing certainty of evidence in the current population (89.3%) is reinforced by the 60.6% prevalence reported in the previous study [5] and the 68% prevalence in the investigation by Gianola et al. [33] Across these previous investigations, the majority ( >90%) of systematic reviews reported assessing the quality, consistency, and/or quantity (precision) of evidence while citing other domains less frequently.

Finally, systematic reviews in the sports science literature were more than twice as likely (26.7%) to include a statement that could be interpreted as a recommendation than in our current investigation of the general and internal medical literature. The lower occurrence of this concern in the current population may be owed to the fact that formal systems for clinical guideline development are more longstanding and commonplace in general and internal medicine than in sports science. In fact, a considerable portion of the included systematic reviews in the current population were developed as part of a larger clinical practice guideline program (e.g., published evidence reports that were developed to inform USPSTF recommendation statements). Especially in these cases, systematic review authors are likely aware that their role is to describe the evidence and evaluate its certainty rather than provide practice recommendations, which is a future step requiring further considerations and input.

### 4.3 Strengths and limitations

To our knowledge, this report is the most extensive review of certainty of evidence assessment in the contemporary medical literature, spanning the past eleven years of systematic reviews published in high‐impact general and internal medicine journals. We adhered to common standards for meta‐epidemiological research, including independent screening of titles, abstracts and full texts by two authors as well as a peer‐to‐peer data extraction review process to improve the accuracy of our reported findings. We decided a priori to exclude reports that included other systematic reviews as part of their search strategy and/or synthesis to ensure a more homogenous population and to simplify our analysis. However, it is possible that our results may differ if these articles were included.

A lack of thorough methodological reporting in our population limits our ability to draw conclusions about which conceptual domains were actually applied by systematic review authors during certainty of evidence assessment, and whether this was done deliberately or due to inadequate familiarity with the certainty of evidence framework being used. Furthermore, we determined the certainty of evidence system used based on the frameworks reported and cited in the methods section of each review. However, in several cases, authors cited one framework (such as the USPSTF system) while applying terminology more similar to those of GRADE or the AHRQ methods guide (high, moderate, low, etc.). Finally, our report does not attempt to describe the quality or accuracy of the way certainty of evidence frameworks are applied in practice, though this matter is ripe for future investigation.

## 5 CONCLUSION

This meta‐epidemiological study describes the state of certainty of evidence assessment in systematic reviews published in high‐impact journals over the past eleven years. Though only approximately one in three systematic reviews in our population assessed certainty of evidence, the frequency of certainty of evidence assessment increased over time. GRADE was the most commonly used framework in our population. Systematic review authors largely adhered to important reporting requirements related to funding, and the majority assessed the certainty of evidence at an appropriate (e.g., outcome) level. Only a small portion of reviews included language that could be construed as a clinical practice recommendation. Finally, systematic review authors did not always report considering each specific domain within a particular certainty of evidence framework. The thoroughness and accuracy of reporting regarding how certainty of evidence frameworks are applied by systematic review authors are unclear, warranting further research and education efforts. Systematic review authors should clearly describe whether and how each specific domain within a certainty of evidence framework was considered to improve the trustworthiness and transparency of their report.

## AUTHOR CONTRIBUTIONS

**Madelin R Siedler**: Conceptualization; Data curation; Formal analysis; Methodology; Project administration; Writing—original draft. **Neha Tangri**: Data curation; Writing—review and editing. **Leena AlShenaiber**: Data curation; Writing—review and editing. **Tejanth Pasumarthi**: Data curation; Writing—review and editing. **Faisal Shaukat Ali**: Data curation; Writing—review and editing. **Volf Gaby**: Data curation; Writing—review and editing. **Katie N Harris**: Data curation; Writing—review and editing. **Yngve Falck‐Ytter**: Writing—review and editing. **Reem A Mustafa**: Writing—review and editing. **Shahnaz Sultan**: Writing—review and editing. **Philipp Dahm**: Writing—review and editing. **M Hassan Murad**: Conceptualization; Methodology; Writing—review and editing. **Rebecca L Morgan**: Conceptualization; Methodology; Writing—review and editing.

## CONFLICT OF INTEREST STATEMENT

The authors declare no financial conflicts of interest. MRS, YFY, RAM, SS, PD, RLM, and MHM are members of the U.S. GRADE Network. MRS, NT, and VG are contractors of the Evidence Foundation and receive direct payment for work completed. All other authors declare no conflicts of interest.

## PEER REVIEW

The peer review history for this article is available at https://www.webofscience.com/api/gateway/wos/peer-review/10.1002/cesm.70014.

## Supporting information

Supporting information.

## Data Availability

The datasets during and/or analyzed during the current study available from the corresponding author on reasonable request.
