# Supplementary material for: Certainty of evidence assessment in high‐impact medical journals: A meta‐epidemiological survey
Source: Cochrane Evid Synth Methods. 2025 Mar 19;3(2):e70014. doi: 10.1002/cesm.70014 (PMC12442678; doi:10.1002/cesm.70014)
Supplement: Supplementary file 1 — Supporting information. [file CESM-3-e70014-s001.docx]

**Appendix 1. Search Strategy**

First search (run 24 January 2023):

("Lancet"[jour] OR "N Engl J Med"[jour] OR "JAMA"[jour] OR "BMJ"[jour] OR "Nat Rev Dis Primers"[jour] OR "Ann Intern Med"[jour] OR "JAMA Intern Med"[jour] OR "J Travel Med"[jour] OR "Lancet Digit Health"[jour] OR "Mil Med Res"[jour]) AND ("Systematic Review"[Publication Type] OR "systematic review"[tiab] OR "meta-analysis" [Publication Type] OR "meta-analysis" [tiab]) AND (("2013/01/24"[Date - Publication] : "2023/01/23"[Date - Publication]))

First update (run 24 July 2023):

("Lancet"[jour] OR "N Engl J Med"[jour] OR "JAMA"[jour] OR "BMJ"[jour] OR "Nat Rev Dis Primers"[jour] OR "Ann Intern Med"[jour] OR "JAMA Intern Med"[jour] OR "J Travel Med"[jour] OR "Lancet Digit Health"[jour] OR "Mil Med Res"[jour]) AND ("Systematic Review"[Publication Type] OR "systematic review"[tiab] OR "meta-analysis" [Publication Type] OR "meta-analysis" [tiab]) AND (("2023/01/24"[Date - Publication] : "2023/07/23"[Date - Publication]))

Second update (run 24 January 2024):

("Lancet"[jour] OR "N Engl J Med"[jour] OR "JAMA"[jour] OR "BMJ"[jour] OR "Nat Rev Dis Primers"[jour] OR "Ann Intern Med"[jour] OR "JAMA Intern Med"[jour] OR "J Travel Med"[jour] OR "Lancet Digit Health"[jour] OR "Mil Med Res"[jour]) AND ("Systematic Review"[Publication Type] OR "systematic review"[tiab] OR "meta-analysis" [Publication Type] OR "meta-analysis" [tiab]) AND (("2023/07/24"[Date - Publication] : "2024/01/23"[Date - Publication]))
